# Drug Repurposing of Voglibose, a Diabetes Medication for Skin Health

**DOI:** 10.3390/ph18020224

**Published:** 2025-02-07

**Authors:** Hyeon-Mi Kim, Chang-Gu Hyun

**Affiliations:** Jeju Inside Agency and Cosmetic Science Center, Department of Chemistry and Cosmetics, Jeju National University, Jeju 63243, Republic of Korea; gusal1388@naver.com

**Keywords:** B16F10, drug repurposing, melanogenesis, signaling pathways, voglibose

## Abstract

**Background/Objectives:** Voglibose, an α-glucosidase inhibitor commonly prescribed to manage postprandial hyperglycemia in diabetes mellitus, demonstrates potential for repurposing as an anti-melanogenic agent. This study aims to explore the inhibitory effects of voglibose on melanogenesis and elucidate its molecular mechanisms, highlighting its possible applications in treating hyperpigmentation disorders. **Methods:** The anti-melanogenic effects of voglibose were investigated using B16F10 melanoma cells. Cell viability, melanin content, and tyrosinase activity were assessed following voglibose treatment. Western blot analysis was performed to examine changes in melanogenic proteins and transcription factors. The role of signaling pathways, including PKA/CREB, MAPK, PI3K/AKT, and GSK3β/β-Catenin, was analyzed. Primary human skin irritation tests were conducted to evaluate the topical safety of voglibose. **Results:** Voglibose significantly reduced melanin synthesis and tyrosinase activity in B16F10 cells in a dose-dependent manner. Western blot analysis revealed decreased expression of MITF, TRP-1, and TRP-2, indicating the inhibition of melanogenesis. Voglibose modulated key signaling pathways, including the suppression of PKA/CREB, MAPK, and AKT activation, while restoring GSK3β activity to inhibit β-catenin stabilization. Human skin irritation tests confirmed voglibose’s safety for topical application, showing no adverse reactions at 50 and 100 μM concentrations. **Conclusions:** Voglibose demonstrates anti-melanogenic properties through the modulation of multiple signaling pathways and the inhibition of melanin biosynthesis. Its safety profile and efficacy suggest its potential as a repurposed drug for managing hyperpigmentation and advancing cosmeceutical applications.

## 1. Introduction

Drug repositioning, also referred to as drug repurposing, has gained prominence as an innovative strategy to identify new therapeutic applications for existing drugs. This approach offers a significant advantage by reducing the time and costs associated with drug development. While bringing a novel chemical entity to market typically demands an average investment of USD 2–3 billion and 10–17 years, drug repositioning achieves comparable outcomes with approximately USD 300 million invested and within 6.5 years. Moreover, the well-established safety profiles of existing drugs significantly lower the risk of clinical failure [1,2,3]. Several successful cases underscore the transformative potential of drug repositioning. Sildenafil (Viagra), originally developed as an antihypertensive agent, was repurposed for erectile dysfunction, achieving worldwide acclaim. Minoxidil, initially designed for hypertension, was later found effective in treating male pattern baldness due to its hypertrichosis side effect. Similarly, baricitinib, initially approved for rheumatoid arthritis, was subsequently used to treat alopecia areata, expanding its therapeutic indications [4,5,6].

In dermatology and skincare, drug repositioning has also proven highly effective. Thalidomide, originally developed as a sedative, is now used for multiple myeloma and skin lesions caused by leprosy. Minocycline, an antibiotic with anti-inflammatory properties, is widely used for treating acne and other inflammatory skin conditions. Methotrexate, developed as an anticancer agent, has been repurposed to treat autoimmune diseases such as psoriasis and rheumatoid arthritis [7,8,9]. The potential of drug repositioning is particularly evident in developing skin-whitening agents and treatments for pigmentation disorders. These disorders arise from abnormalities in melanin synthesis or distribution, which drug repositioning can effectively address by leveraging the established safety and efficacy profiles of existing drugs. For example, nilotinib, sorafenib, and ICG-001 promote melanin synthesis, while 5-iodotubercidin inhibits it. These discoveries highlight drug repositioning as a powerful tool for managing pigmentation disorders and advancing dermatological therapies [10]. In the cosmeceuticals industry, which combines the esthetic benefits of cosmetics with the functional efficacy of pharmaceuticals, drug repositioning plays a vital role. This approach addresses various concerns, including skin whitening, wrinkle reduction, and hair growth promotion, by targeting specific molecular mechanisms and pathways of existing drugs. This strategy not only accelerates development but also minimizes costs and timelines, offering innovative solutions [11,12,13].

Our research group has actively explored the potential of drug repositioning and natural compounds in therapeutic development. For instance, acenocoumarol, an anticoagulant, demonstrated anti-inflammatory effects by inhibiting NF-κB and mitogen-activated protein kinase (MAPK) signaling pathways, reducing pro-inflammatory mediators such as nitric oxide (NO), prostaglandin E_2_ (PGE_2_), and cytokines (TNF-α, IL-6). Miglitol, an antidiabetic drug, exhibited skin-whitening effects by suppressing melanin synthesis through PKA, MAPK, and GSK3β/β-catenin pathways. Similarly, imperatorin, a natural compound, showed promise as a preventive and therapeutic agent for pigmentary disorders by inhibiting melanogenesis. Nojirimycin also displayed significant anti-inflammatory activity through NF-κB and MAPK pathway inhibition, highlighting its potential for dermatological applications [14,15,16,17].

**Figure 1:**
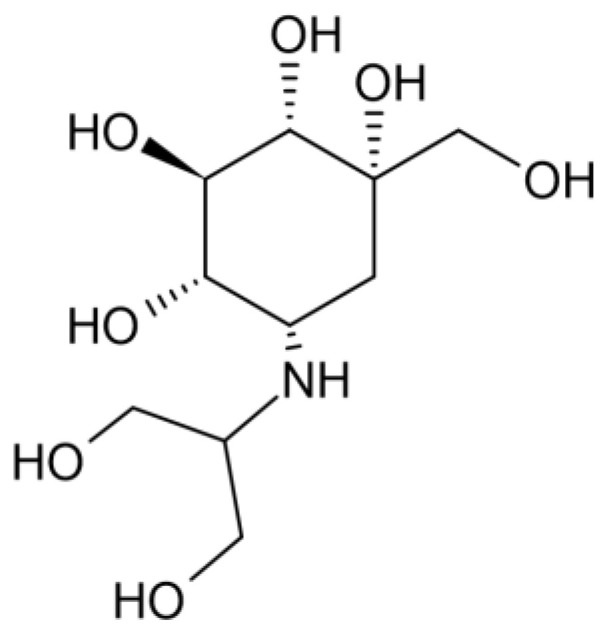
The structure of voglibose.

Voglibose (Figure 1) is a pseudo-sugar derivative of valiolamine, characterized by a unique structure that allows it to inhibit α-glucosidase enzymes by mimicking the transition state of the substrate. Originally developed for the management of type 2 diabetes, voglibose effectively reduces postprandial glucose levels by delaying carbohydrate digestion [18,19,20].

Beyond its role in glucose regulation, voglibose has been reported to exhibit anti-inflammatory and antioxidative properties, which are crucial factors in maintaining skin health and mitigating hyperpigmentation-related conditions. As a representative antidiabetic drug with inhibitory activity toward human α-glucosidase, voglibose has been shown to block the proper N-glycan modification of tyrosinase, leading to a dramatic reduction in tyrosinase protein levels by altering its stability and subsequently decreasing melanin production [21,22,23,24]. Recent studies have revealed that α-glucosidase inhibitors can regulate pigmentation by modulating key melanogenic enzymes such as tyrosinase, TRP-1, and TRP-2 [15]. This suggests that the inhibition of glycosylation processes is directly linked to skin brightening and a reduction in pigmentation, highlighting voglibose as a promising candidate for pigmentation disorder treatment.

In this study, we systematically investigated the effects of voglibose on melanin biosynthesis using the B16F10 melanoma cell model. Specifically, we analyzed its regulatory mechanisms acting on key melanogenic enzymes, including tyrosinase, and various signaling pathways involved in melanogenesis to elucidate its mode of action and assess its potential application for skin pigmentation control.

## 2. Results and Discussion

### 2.1. Cell Viability Assessment

**Figure 2:**
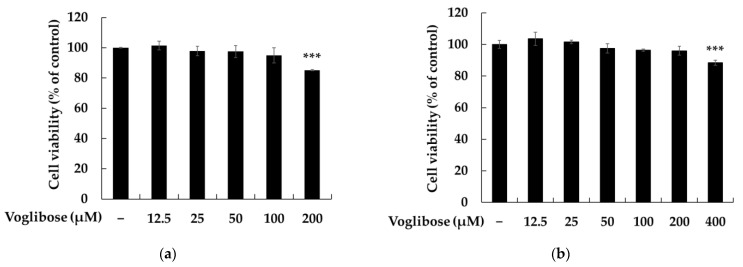

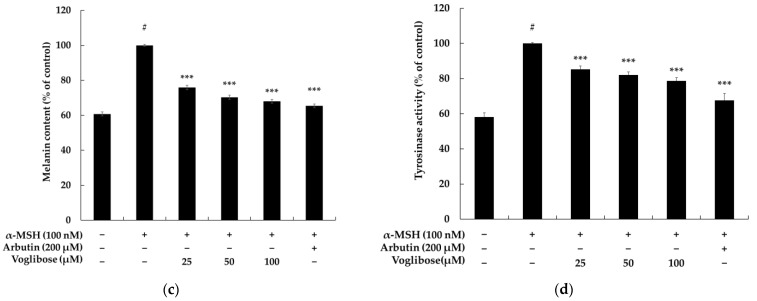
The effects of voglibose on cell viability, melanin production, and tyrosinase activity in B16F10 cells. Cell viability was assessed under two conditions. In (**a**), cells were plated at 1.0 × 10^5^ cells/well and treated with voglibose for 24 h. In (**b**), cells were plated at 2.0 × 10^4^ cells/well and treated for 72 h. Cell viability was expressed as a percentage relative to untreated cells. Voglibose treatment reduced melanin content (**c**) and tyrosinase activity (**d**) in B16F10 cells. α-melanocyte-stimulating hormone (α-MSH) was used as a negative control to induce melanogenesis, and arbutin was used as a positive control to inhibit it. A cell viability assay was performed in triplicate (*n* = 3), and all data are presented as the mean ± standard deviation (SD) from at least three independent experiments. Statistical significance was denoted as *** *p* < 0.001 vs. the untreated control or the α-MSH-only group and # *p* < 0.001 compared to the untreated group.

In the development of functional ingredients using cell-based assays, assessing cell viability is a critical step to avoid misinterpretation of results. Reduced cell viability can lead to nonspecific effects that may be misconstrued as functional activity. To accurately evaluate the safety and efficacy of an ingredient, cell viability data must be utilized to distinguish between cytotoxic effects and genuine physiological outcomes, ensuring the scientific validation of functional ingredients [25,26]. To establish experimental conditions for evaluating the melanin-inhibitory effects of voglibose, cell viability was assessed using the MTT assay in B16F10 cells under different plating densities and culture durations. Cells were cultured with voglibose for 24 h and 72 h, and viability was analyzed under two distinct conditions: plating 1 × 10^5^ cells/well with 200 μM voglibose for 24 h (Figure 2a) and plating 2 × 10^4^ cells/well with 400 μM voglibose for 72 h (Figure 2b). The results showed a significant effect on cell viability under both conditions, with a viability of 85.14% at 200 μM after 24 h and 96.06% at 400 μM after 72 h. The differences in cell viability indicate that both plating density and culture duration influenced cellular response to voglibose. A higher initial cell density (1 × 10^5^ cells/well) with a shorter culture time (24 h) led to a more pronounced decrease in viability, whereas a lower plating density (2 × 10^4^ cells/well) with an extended culture period (72 h) resulted in relatively stable viability.

These findings highlight the importance of considering plating density and incubation time when evaluating the effects of voglibose on cell viability. To ensure the validity of efficacy testing in B16F10 cells, voglibose concentrations were set to 100 μM or lower to prevent potential cytotoxic effects.

### 2.2. Effects of Voglibose on Melanin Synthesis and Tyrosinase Activity in B16F10 Cells

B16F10 cells, a murine melanoma cell line, are widely used for melanin synthesis studies due to their key regulatory pathways, including MITF, tyrosinase, TRP-1, and TRP-2. Cell-based models are cost-effective, reproducible, and useful for studying molecular mechanisms [27,28,29]. Melanin content and tyrosinase activity serve as crucial indicators for evaluating melanin synthesis inhibitors. Since tyrosinase regulates the rate-limiting step in melanin production, assessing its inhibition provides insights into the mechanism of action of potential inhibitors [30,31,32]. Thus, the B16F10 cell model is a reliable platform for investigating the regulatory effects of samples on melanin synthesis. Accordingly, in this study, we investigated the effect of voglibose on melanin synthesis and tyrosinase activity induced by α-MSH (100 nM) using the B16F10 cell model. As shown in Figure 2, α-MSH treatment increased melanin content by 39.26% compared to the untreated control. In contrast, arbutin (200 μM), used as a positive control, reduced melanin content by 34.53%. Voglibose treatment at concentrations of 25, 50, and 100 μM reduced melanin content by 24.05%, 29.65%, and 32.00%, respectively. Similarly, α-MSH treatment increased tyrosinase activity by 41.95% compared to the control. Arbutin (200 μM) decreased tyrosinase activity by 32.32%, while voglibose at concentrations of 25, 50, and 100 μM reduced tyrosinase activity by 14.70%, 18.01%, and 21.35%, respectively. These results indicate that voglibose inhibits both α-MSH-induced melanin synthesis and tyrosinase activity in a dose-dependent manner, suggesting its potential as an effective melanin synthesis inhibitor.

### 2.3. The Effects of Voglibose on Melanogenic Proteins and the Expression of the Transcription Factor MITF in B16F10 Cells

Melanogenesis is a critical physiological process responsible for melanin pigment synthesis, playing a key role in pigmentation of the skin, hair, and eyes. This process is regulated by major proteins, including tyrosinase, TRP-1, and TRP-2. Tyrosinase is a key enzyme catalyzing the initial steps of melanin biosynthesis by oxidizing L-tyrosine to L-DOPA and converting it into dopaquinone. As the rate-limiting enzyme in melanin biosynthesis, tyrosinase directly influences the rate of melanin production. TRP-1 stabilizes melanin synthesis and plays an essential role in regulating the ratio of eumelanin (brown and black pigments) to pheomelanin (yellow and red pigments). Additionally, TRP-1 maintains the structural stability of melanosomes and contributes to the final structural formation of melanin. TRP-2 functions in the intermediate steps of melanin biosynthesis by converting dopachrome to 5,6-dihydroxyindole-2-carboxylic acid (DHICA), preventing oxidative damage to melanin, and providing antioxidant effects to enhance melanin stability [33,34,35].

**Figure 3:**
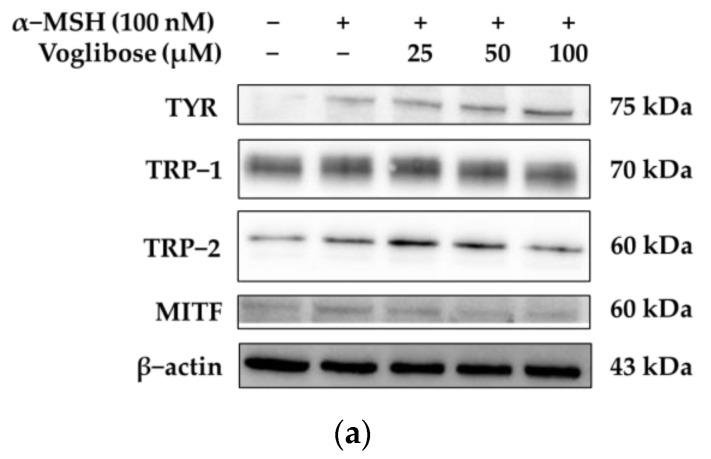

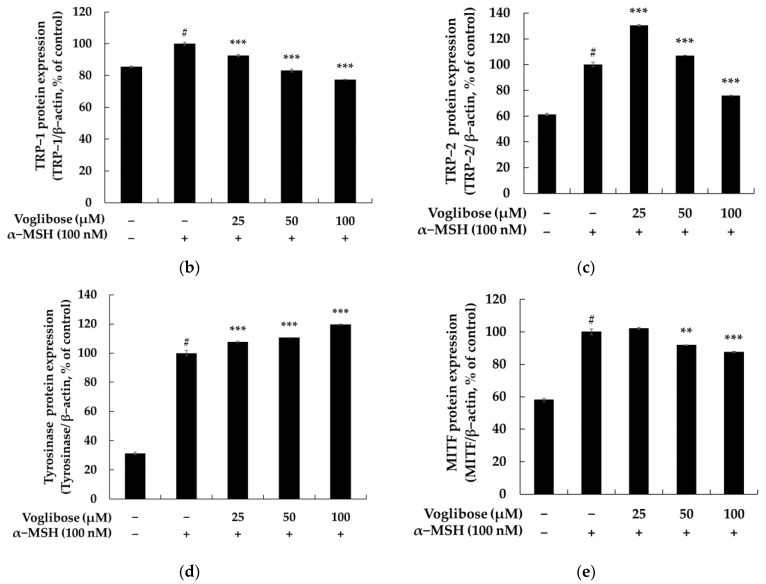
The effects of voglibose on melanogenic proteins and the expression of the transcription factor MITF in B16F10 cells. (**a**) The Western blot results illustrating the expression changes of tyrosinase (TYR), TRP-1, TRP-2, and MITF, a key transcription factor regulating melanogenesis. (**b**–**e**) Quantitative graphs of TRP-1, TRP-2, tyrosinase, and MITF protein expression visually depicting the effects of voglibose treatment on protein expression. α-MSH (100 nM) was used to induce melanogenesis, and statistical significance is denoted as # *p* < 0.001 compared to the untreated control and ** *p* < 0.01 or *** *p* < 0.001 compared to the α-MSH-only group. Data are expressed as the mean ± standard deviation (SD) from at least three independent experiments, each performed in triplicate, and analyzed using ImageJ software (version 1.54k, NIH, Bethesda, MD, USA).

In this study, the effects of voglibose on melanin content reduction, tyrosinase activity inhibition, and the expression of tyrosinase, TRP-1, and TRP-2 proteins were investigated. Western blot analysis was performed, and the results are summarized as follows: In the α-MSH (100 nM) treatment group, TRP-1 protein expression increased by 11.99%. However, when voglibose was administered at concentrations of 25, 50, and 100 μM, TRP-1 protein expression decreased by 7.45%, 16.84%, and 22.46%, respectively, compared to α-MSH treatment. For TRP-2, α-MSH (100 nM) treatment led to a 38.57% increase in protein expression. When treated with 25 μM voglibose, TRP-2 expression increased by 30.47% compared to α-MSH, whereas at 100 μM, it showed a 24.08% decrease. Interestingly, the results for tyrosinase protein expression were contrary to the initial hypothesis. Tyrosinase protein expression increased in a concentration-dependent manner with voglibose treatment, showing increases of 7.69%, 10.65%, and 19.60% at concentrations of 25, 50, and 100 μM, respectively (Figure 3). However, despite the increased protein expression, tyrosinase activity was inhibited. This suggests that voglibose may interfere with post-translational modifications (e.g., glycosylation) or activation processes of tyrosinase, impairing its functional activation.

The paradoxical effects of voglibose on tyrosinase activity inhibition and protein expression increase are likely related to its glycoprotein characteristics. Tyrosinase activity, stability, and proper transport to melanosomes are regulated by N-linked glycosylation. Efficient glycosylation enables tyrosinase to maintain its active state and effectively regulate the rate-limiting step of melanin biosynthesis. In contrast, insufficient or incomplete glycosylation may lead to the accumulation or degradation of inactive tyrosinase [36,37].

As an α-glucosidase inhibitor, voglibose may reduce the supply of monosaccharides (e.g., glucose-6-phosphate, mannose) required for glycosylation, thereby decreasing tyrosinase glycosylation efficiency. This can hinder tyrosinase activation and induce the accumulation of inactive enzymes [24,38,39]. Consequently, even with normal synthesis of the tyrosinase protein, reduced enzyme activity may lead to decreased melanin production. Tyrosinase relies heavily on the endoplasmic reticulum (ER) for proper protein folding and post-translational modifications. Metabolic changes induced by voglibose may cause ER stress, potentially disrupting tyrosinase folding or impairing the ER quality control system, such as chaperone proteins. This can lead to the accumulation of inactive tyrosinase in the ER or its degradation via the ER-associated degradation (ERAD) pathway [40,41]. As a redox-sensitive enzyme, tyrosinase requires an appropriate oxidative environment for activation. Voglibose-induced α-glucosidase inhibition may alter cellular metabolism and affect NADPH and GSH (glutathione) levels, disrupting redox balance. This could result in reduced tyrosinase activity and decreased melanin synthesis [42,43]. Additionally, voglibose may exhibit nonspecific effects at high concentrations, indirectly affecting glycosylation-related enzymes or protein quality control pathways, further impairing tyrosinase glycosylation and transport [44]. In conclusion, voglibose induces changes in cellular metabolism and glycosylation processes through α-glucosidase inhibition, leading to an imbalance between tyrosinase protein production and activation.

MITF is a key transcription factor that regulates melanin biosynthesis in melanocytes and plays a pivotal role in melanogenesis. MITF directly activates the transcription of melanin biosynthesis-related genes such as tyrosinase, TRP-1, and TRP-2, thereby promoting the melanin production process. Additionally, MITF is essential for maintaining melanocyte differentiation, proliferation, and survival, as well as regulating melanosome formation. By responding to external stimuli such as UV exposure and oxidative stress, MITF modulates melanin biosynthesis and provides robust skin protective functions. Furthermore, MITF contributes to maintaining cellular homeostasis by participating in pathways related to energy metabolism within melanocytes [45,46,47,48].

Given these critical functions of MITF, excessive melanin production has been identified as a major cause of various skin disorders, including melasma, freckles, lentigines, and hyperpigmentation associated with melanin over-accumulation. Therefore, strategies aimed at reducing melanin synthesis by suppressing MITF expression and activity may represent an essential approach for developing effective melanogenesis inhibitors [49,50].

In this study, we analyzed the effects of voglibose on MITF expression and activity, and we performed Western blot analysis to elucidate the relationship between melanin production inhibition and the suppression of TRP-1 and TRP-2 protein synthesis. As shown in Figure 3a,d, α-MSH (100 nM) treatment increased MITF expression by 41.96%. However, treatment with voglibose at concentrations of 50 μM and 100 μM resulted in a reduction in MITF expression by 8.19% and 12.46%, respectively, compared to α-MSH (100 nM) treatment (Figure 3a,d). These results suggest that voglibose inhibits the MITF signaling pathway, thereby contributing to the suppression of TRP-1 and TRP-2 protein synthesis.

### 2.4. Exploring the Anti-Melanogenic Potential of Voglibose Through Signal Pathway Modulation

Melanogenesis is a vital physiological process responsible for the pigmentation of the epidermis, follicles, and irises, achieved through melanin synthesis regulated by intricate signaling pathway interactions. This process is primarily mediated by the α-MSH/melanocortin 1 receptor (MC1R) pathway and is closely associated with pathways such as protein kinase A (PKA)/cAMP response element-binding protein (CREB), glycogen synthase kinase (GSK)3β/β-catenin, AKT, MAPKs, and p53 [51,52,53]. These pathways regulate the expression and activity of MITF, which controls the transcription of melanogenic genes (tyrosinase, TRP-1, TRP-2) and promotes or inhibits melanosome formation. Studies on the mechanisms regulating melanogenesis provide a foundation for treating hyperpigmentation disorders, such as melasma and freckles, caused by excessive melanin production, as well as for developing effective melanin inhibitors. In particular, understanding the molecular mechanisms centered on MITF-mediated signaling pathways can help identify novel therapeutic targets and develop functional materials to modulate melanin synthesis. This study explores the mechanisms of melanogenesis inhibition, focusing on these signaling pathways and the role of MITF [54,55,56].

**Figure 4:**
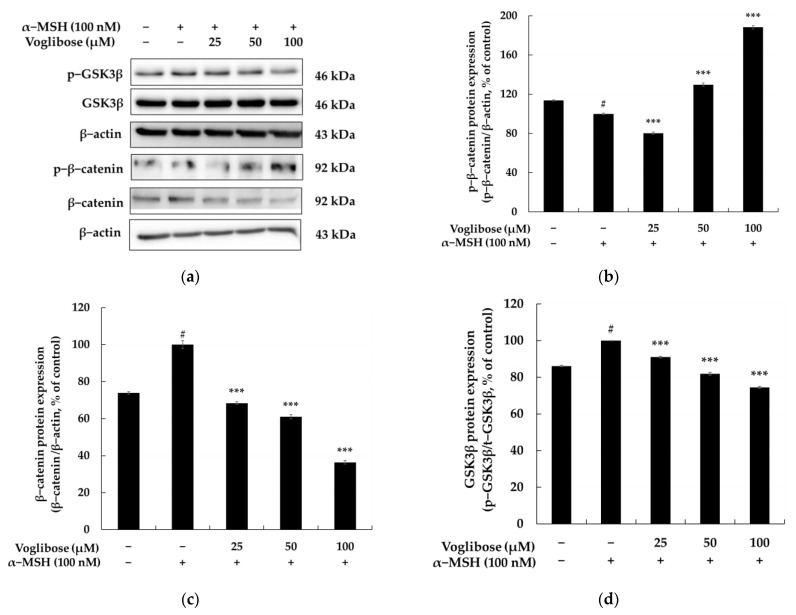
The relationship between the anti-melanogenic effects of voglibose and the Wnt/β-catenin signaling pathway in B16F10 cells. (**a**) The Western blot results for phosphorylated GSK3β (p-GSK3β), total GSK3β, phosphorylated β-catenin (p-β-catenin), and total β-catenin, normalized to β-actin as the loading control. (**b**–**d**) Quantitative analyses of protein expression levels, highlighting the dose-dependent effects of voglibose. Voglibose treatment increased p-β-catenin expression (**b**) while decreasing total β-catenin levels (**c**) and reducing p-GSK3β expression (**d**). These results indicate that voglibose restores GSK3β activity, promotes β-catenin degradation, and modulates the Wnt/β-catenin signaling pathway. α-MSH (100 nM) was used to induce melanogenesis, and statistical significance is denoted as # *p* < 0.001 compared to the untreated control and *** *p* < 0.001 compared to the α-MSH-only group. Data are expressed as the mean ± SD from at least three independent triplicate experiments and analyzed using ImageJ software version 1.54k (NIH, Bethesda, MD, USA).

Figure 4 illustrates the results of evaluating the involvement of the GSK3β/β-catenin signaling pathway in the inhibitory effects of voglibose on melanin biosynthesis. The GSK3β/β-catenin signaling pathway plays a pivotal role in melanogenesis, where the activation of GSK3β induces β-catenin degradation, suppressing MITF expression and ultimately reducing melanin production. Conversely, the inhibition of GSK3β stabilizes β-catenin, leading to its nuclear translocation and promoting MITF transcription, thereby activating melanin synthesis [57,58,59]. Treatment with α-MSH (100 nM) decreased p-β-catenin protein expression (p-β-catenin/β-actin, % of control) by 13.68% compared to the control group, activating β-catenin and enhancing melanogenesis. In contrast, treatment with voglibose at concentrations of 50 and 100 μM increased p-β-catenin protein expression by 29.73% and 88.27%, respectively, compared to the α-MSH-treated group, demonstrating the inhibition of β-catenin activity. Additionally, α-MSH (100 nM) treatment increased β-catenin protein expression (β-catenin/β-actin, % of control) by 26.05% compared to the control, promoting melanogenesis. However, treatment with voglibose at concentrations of 25, 50, and 100 μM reduced β-catenin protein expression by 31.69%, 39.00%, and 63.59%, respectively, compared to the α-MSH-treated group, indicating that voglibose effectively inhibits melanogenesis by suppressing β-catenin expression. The phosphorylation status of GSK3β, reflected by the ratio of p-GSK3β/t-GSK3β (% of control), increased by 19.29% following α-MSH (100 nM) treatment compared to the control group, indicating GSK3β inhibition and β-catenin stabilization. However, voglibose treatment at 50 and 100 μM concentrations decreased GSK3β phosphorylation levels by 5.67% and 6.33%, respectively, compared to the α-MSH-treated group. This finding suggests that voglibose restores GSK3β activity, promotes β-catenin degradation, and suppresses melanogenesis.

**Figure 5:**
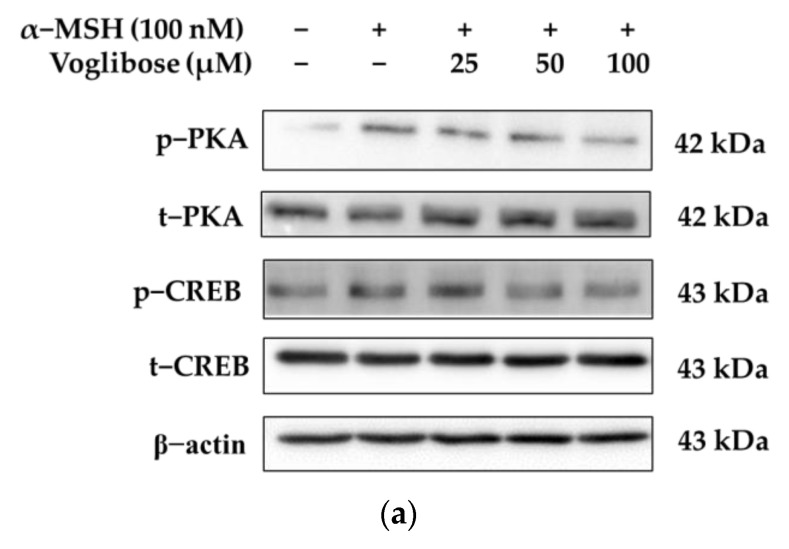

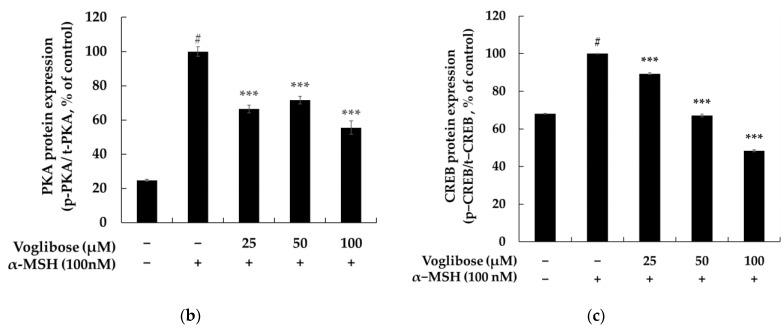
The relationship between the anti-melanogenic effects of voglibose and the PKA/CREB signaling pathway in B16F10 cells. (**a**) The Western blot results for phosphorylated PKA (p-PKA), total PKA (t-PKA), phosphorylated CREB (p-CREB), and total CREB (t-CREB), with β-actin used as a loading control. (**b**,**c**) Quantitative analyses of protein expression levels, demonstrating the dose-dependent effects of voglibose. Voglibose treatment reduced p-PKA expression (**b**) and p-CREB expression (**c**), indicating its inhibitory effect on the PKA/CREB pathway. Statistical significance is denoted as *# p* < 0.001 compared to the untreated control and *** *p* < 0.001 compared to the α-MSH-only group. Data are expressed as the mean ± SD from at least three independent triplicate experiments and analyzed using ImageJ software (version 1.54k; NIH, Bethesda, MD, USA).

Figure 5 illustrates the results of evaluating the involvement of the PKA/CREB signaling pathway in the inhibitory effects of voglibose on melanin biosynthesis. The PKA/CREB signaling pathway is a critical regulatory mechanism in melanogenesis. α-MSH binds to MC1R, inducing cAMP production, which activates PKA. Activated PKA phosphorylates CREB, promoting the transcription of MITF and inducing the expression of melanogenic enzymes [60,61,62,63].

Treatment with α-MSH (100 nM) increased PKA phosphorylation (p-PKA/t-PKA, % of control) by 75.26% compared to the control group, promoting melanogenesis through PKA pathway activation. In contrast, voglibose treatment at concentrations of 25, 50, and 100 μM reduced PKA phosphorylation by 33.49%, 28.43%, and 45.57%, respectively, compared to the α-MSH-treated group. These findings indicate that voglibose can regulate melanogenesis by inhibiting PKA phosphorylation, thereby suppressing the PKA pathway.

Additionally, α-MSH (100 nM) treatment increased CREB phosphorylation (p-CREB/t-CREB, % of control) by 31.95% compared to the control group, promoting MITF transcription through CREB activation. Conversely, voglibose treatment at concentrations of 25, 50, and 100 μM reduced CREB phosphorylation by 10.68%, 32.98%, and 51.64%, respectively, compared to the α-MSH-treated group. These results suggest that voglibose contributes to the inhibition of melanogenesis by suppressing CREB phosphorylation, thereby downregulating MITF transcription. These findings demonstrate that voglibose possesses a molecular mechanism that inhibits the PKA/CREB signaling pathway activated by α-MSH in a concentration-dependent manner, reducing MITF expression and melanin biosynthesis.

**Figure 6:**
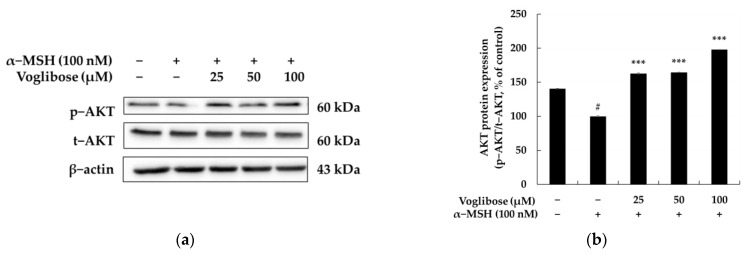
The relationship between the anti-melanogenic effects of voglibose and the AKT signaling pathway in B16F10 cells. (**a**) The Western blot results for phosphorylated AKT (p-AKT) and total AKT (t-AKT), with β-actin used as a loading control. (**b**) Quantitative analysis of p-AKT expression, demonstrating the dose-dependent effects of voglibose treatment. Voglibose significantly reduced p-AKT expression, indicating its inhibitory effect on the AKT signaling pathway. α-MSH (100 nM) was used to induce melanogenesis, and statistical significance is denoted as # *p* < 0.001 compared to the untreated control and *** *p* < 0.001 compared to the α-MSH-only group. Data are expressed as the mean ± SD from at least three independent triplicate experiments and analyzed using ImageJ software version 1.54k (NIH, Bethesda, MD, USA).

Figure 6 illustrates the results of evaluating the involvement of the AKT signaling pathway in the inhibitory effects of voglibose on melanin biosynthesis. AKT is a key signaling molecule in melanogenesis, regulating MITF expression and the activation of melanogenic enzymes. Activation of AKT inhibits GSK3β, promoting the stabilization of β-catenin, which in turn induces MITF transcription and stimulates melanin synthesis [64,65,66]. Therefore, inhibition of the AKT signaling pathway can effectively reduce melanin production. Treatment with α-MSH (100 nM) increased AKT phosphorylation by 31.95% compared to the control group, activating melanogenesis. In contrast, treatment with voglibose at concentrations of 25, 50, and 100 μM reduced AKT phosphorylation by 11.68%, 32.98%, and 51.64%, respectively, compared to the α-MSH-treated group. These findings suggest that voglibose suppresses MITF activation by inhibiting the AKT signaling pathway, consequently reducing the expression of melanogenic enzymes. The downregulation of the AKT signaling pathway serves as a critical mechanism for inhibiting melanogenesis, demonstrating the potential of voglibose as an effective agent for regulating melanin biosynthesis.

**Figure 7:**
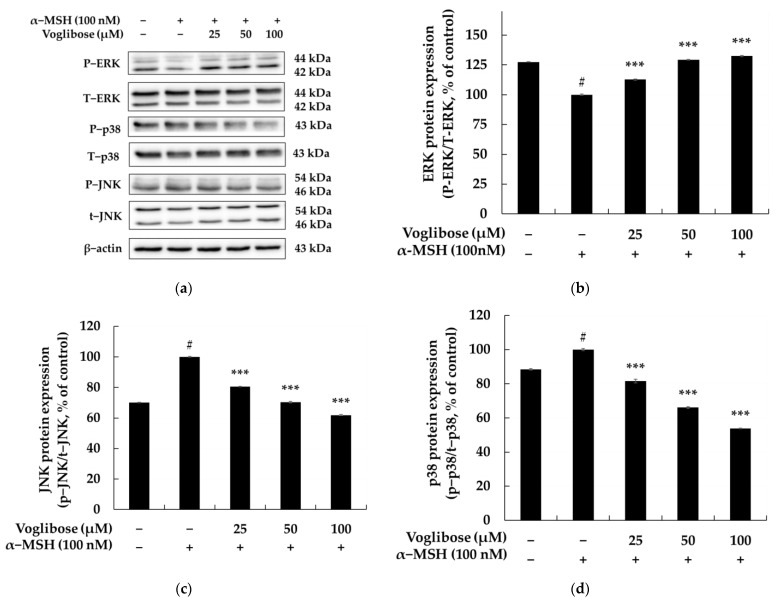
The relationship between the anti-melanogenic effects of voglibose and the MAPK signaling pathway in B16F10 cells. (**a**) The Western blot results for phosphorylated and total ERK, p38, and JNK proteins, normalized to β-actin as the loading control. (**b**–**d**) Quantitative analyses of protein expression levels, demonstrating the dose-dependent effects of voglibose treatment. Voglibose treatment increased p-ERK expression (**b**) while significantly decreasing p-JNK (**c**) and p-p38 (**d**) expression levels, indicating its modulatory effects on the MAPK signaling pathway. α-MSH (100 nM) was used to induce melanogenesis, and statistical significance is denoted as # *p* < 0.001 compared to the untreated control and *** *p* < 0.001 compared to the α-MSH-only group. Data are expressed as the mean ± SD from at least three independent triplicate experiments and analyzed using ImageJ software (version 1.54k, NIH, Bethesda, MD, USA).

Figure 7 presents the results of evaluating the effects of voglibose on the phosphorylation of major MAPK proteins (ERK, JNK, p38) in B16F10 cells stimulated with α-MSH, highlighting its relationship with melanin biosynthesis inhibition. The MAPK signaling pathway is a key mechanism regulating melanin production, activated in melanocytes by external stimuli such as α-MSH. This pathway regulates MITF and the expression of melanogenic enzymes and consists of three sub-pathways: ERK, JNK, and p38. ERK plays an inhibitory role in melanin synthesis by promoting the degradation of MITF when activated (phosphorylated), thereby reducing the expression of melanogenic enzymes. Conversely, ERK inhibition stabilizes MITF, promoting melanin synthesis. JNK acts as a pro-melanogenic pathway, increasing MITF transcriptional activity upon activation, which induces the expression of melanogenic enzymes. This pathway is activated by stress signals or UV radiation, with increased JNK phosphorylation enhancing melanin synthesis and contributing to skin hyperpigmentation. Similarly, the p38 pathway promotes melanin synthesis by stabilizing MITF and increasing the expression of melanogenic enzymes [67,68,69,70]. Environmental stress, such as UV exposure, activates p38, and its phosphorylation enhances melanin production and skin pigmentation. The MAPK pathway functions through the interplay of ERK, JNK, and p38, where ERK suppresses melanin synthesis, while JNK and p38 promote it. This balance regulates melanogenesis and impacts conditions such as skin hyperpigmentation, vitiligo, and freckles. Modulating ERK, JNK, and p38 pathways serves as a strategic approach for developing skin-whitening agents and treatments for pigmentation disorders. The experimental results show that α-MSH treatment reduced ERK phosphorylation by 27.45% while increasing JNK and p38 phosphorylation by 29.85% and 11.56%, respectively. However, treatment with voglibose at 25, 50, and 100 μM increased ERK phosphorylation by 12.70%, 29.31%, and 32.46%, respectively, compared to the α-MSH-treated group. Meanwhile, voglibose reduced JNK phosphorylation by 19.61%, 29.61%, and 38.10% and p38 phosphorylation by 18.38%, 33.89%, and 46.09%, respectively. These findings indicate that voglibose inhibits the JNK and p38 signaling pathways activated by α-MSH, effectively suppressing melanin biosynthesis. The reduced phosphorylation of JNK and p38 likely plays a crucial role in downregulating the expression of melanogenic enzymes. In contrast, the increased ERK phosphorylation observed with voglibose treatment suggests an alternative mechanism by which voglibose promotes the degradation of MITF via ERK activation, thereby inhibiting melanin synthesis.

### 2.5. Human Skin Irritation Study of Voglibose

Ensuring skin safety is a critical step in the development of functional whitening agents. First, skin safety minimizes the risk of irritation or allergic reactions, which may result from the potent chemical properties of certain whitening ingredients. By establishing safety, products can be delivered to consumers with confidence in their usability. Second, high skin safety enhances the feasibility of long-term product use. Products with proven stability are less likely to cause adverse effects over extended usage periods, contributing to increased consumer satisfaction. Third, products with demonstrated skin safety build trust among consumers, which is essential for creating a positive brand image. Reliable products not only encourage repeat purchases but also foster long-term brand loyalty. Fourth, compliance with safety regulations is a fundamental requirement for cosmetic and skincare products. Securing skin safety ensures adherence to these regulatory standards. Finally, ensuring skin safety supports the effective delivery of whitening benefits without adverse effects on the skin. As such, securing the skin safety of functional whitening agents is a vital component for product success and user satisfaction [71,72,73].

Therefore, in this study, the skin safety and potential of voglibose for topical applications were evaluated through primary human skin irritation tests. Voglibose was applied to designated skin areas at concentrations of 50 and 100 μM for up to 24 h. After the removal of voglibose, the application sites were observed at 20 min and 24 h post-application. Squalene, used as a solvent, served as the negative control. As summarized in Table 1, voglibose was classified as having “no to slight irritation” based on its primary irritation potential on human skin. These findings suggest that voglibose demonstrates minimal irritation, supporting its suitability for topical use.

## 3. Materials and Methods

### 3.1. Reagents and Materials

Voglibose (>98.0%) was purchased from TCI Co., Ltd. (Tokyo, Japan). MTT (3-(4,5-dimethylthiazol-2-yl)-2,5-diphenyltetrazolium bromide) reagent, 10× Tris-Glycine (SDS) buffer, RIPA buffer, DMSO, PBS, and the ECL (Enhanced Chemiluminescence) kit were obtained from Biosesang Inc. (Seongnam, Gyeonggi-do, Republic of Korea). Kojic acid, L-DOPA, protease/phosphatase inhibitor cocktail, and α-MSH were purchased from Sigma-Aldrich Co. LLC (St. Louis, MO, USA). FBS (fetal bovine serum) was acquired from Merck KGaA (Burlington, MA, USA). DMEM (Dulbecco’s Modified Eagle’s Medium) and penicillin–streptomycin (10,000 U/mL) were supplied by Thermo Fisher Scientific Inc., through its Gibco brand (Grand Island, NY, USA).

Primary antibodies included MITF (SC-71588), tyrosinase (SC-20035), TRP-1 (SC-166857), and TRP-2 (SC-74439), all purchased from Santa Cruz Biotechnology, Inc. (Dallas, TX, USA). Antibodies from Cell Signaling Technology, Inc. (Danvers, MA, USA) included CREB (D76D11) Rabbit mAb (4820S), phospho-CREB (Ser133) (87G3) Rabbit mAb (9198S), PKA and phospho-PKA C (Thr197) (D45D3) Rabbit mAb (5661S), β-catenin (D10A8) XP^®^ Rabbit mAb (25362S), phospho-β-catenin (S33/37/T41) Rabbit Ab (9561S), phospho-SAPK/JNK (Thr183/Tyr185) Antibody (9251S), SAPK/JNK Rabbit Ab (9252S), p44/42 MAPK (Erk1/2) Antibody (9102S), P-p44/42 MAPK (T202/Y204) Rabbit Ab (9101S), P-p38 MAPK (T100/Y182) Rabbit Ab (9211S), p38 MAPK Rabbit Ab (9212S), phospho-GSK-3-β (S9) (D3A4) Rabbit mAb (9322S), GSK-3 α/β (D75D3) Rabbit mAb (5676S), Akt Rabbit Ab (9272S), phospho-Akt (S473) Rabbit Ab (9271S), and β-actin Rabbit Antibody (4967S).

Secondary antibodies, Anti-rabbit IgG HRP-linked Antibody (7074S) and Anti-mouse IgG HRP-linked Antibody (7076S), were also obtained from Cell Signaling Technology, Inc. The inhibitors used in this study included PD98059, an ERK inhibitor purchased from Cayman Chemical Company (Ann Arbor, MI, USA), and LY294002, an AKT inhibitor obtained from Cell Signaling Technology, Inc. Phosphorylation sites on proteins, such as phospho-CREB (Ser133), phospho-PKA C (Thr197), phospho-β-catenin (S33/37/T41), phospho-SAPK/JNK (Thr183/Tyr185), P-p44/42 MAPK (T202/Y204), P-p38 MAPK (T100/Y182), and phospho-GSK-3-β (S9), were annotated based on the respective amino acid residues.

### 3.2. Cell Culture

The B16F10 murine melanoma cell line was acquired from the American Type Culture Collection (ATCC) and the Korean Cell Line Bank (KCLB). B16F10 cells were maintained at 37 °C in a humidified atmosphere with 5% CO_2_. The culture medium used was Dulbecco’s Modified Eagle’s Medium (DMEM; Thermo Fisher Scientific Inc., Grand Island, NY, USA) supplemented with 10% fetal bovine serum (FBS) and 1% penicillin/streptomycin (10,000 U/mL).

### 3.3. Cell Viability Assay

Cell viability was evaluated using the MTT assay. B16F10 cells were seeded and cultured in a 24-well plate at a density of 1.5 × 10^4^ cells/well. After 24 h, voglibose was added at various concentrations (12.5, 25, 50, 100, 200, and 400 μM). Following a 72 h incubation period, the MTT reagent was added to each well, and the plate was incubated for less than 4 h at 37 °C. The absorbance was measured at 540 nm using a microplate spectrophotometer (BioTek Epoch, Agilent Technologies, Inc., Winooski, Vermont, USA).

### 3.4. Melanin Content Measurement

B16F10 melanoma cells stimulated to induce melanogenesis were washed twice with cold PBS, and total proteins were extracted using RIPA buffer. The cell lysates were centrifuged at 15,000 rpm for 20 min at −8 °C, and the resulting pellet was separated. The pellet was dissolved in 1 N NaOH containing 10% DMSO and incubated at 80 °C for 10 min. The melanin content was measured at 405 nm using a spectrophotometric microplate reader with a 96-well plate.

### 3.5. Tyrosinase Activity Assay

B16F10 melanoma cells stimulated to induce melanin production were washed twice with PBS, and total proteins were extracted using RIPA buffer. The cell lysates were centrifuged at 15,000 rpm for 20 min at 4 °C, and the total protein concentration in the supernatant was determined using a BCA assay. For the tyrosinase activity assay, the reaction mixture consisted of 80 μL of cell lysate (containing 30 μg/mL total protein) and 20 μL of L-DOPA solution (2 mg/mL in 0.1 M sodium phosphate buffer, pH 6.8) in a 96-well plate. The mixture was incubated at 37 °C, and the production of dopaquinone was measured at 490 nm using a spectrophotometric microplate reader after 2 h.

### 3.6. Western Blot Analysis

B16F10 melanoma cells treated with voglibose (25 to 100 μM) were harvested using RIPA buffer supplemented with a protease inhibitor cocktail. The protein concentration of the cell lysates was determined using a BCA protein assay kit and adjusted to 30 μg/mL. Protein samples were prepared by mixing the lysates with sample buffer (2× Laemmli buffer: 2-mercaptoethanol = 20:1) and heating at 100 °C for 5 min. Proteins were separated using Tris-Glycine SDS-polyacrylamide gel electrophoresis and transferred onto a PVDF membrane. The membrane was blocked with 5% skim milk prepared in TBS-T to prevent nonspecific binding of antibodies. After blocking, the membrane was washed with TBS-T and incubated with primary antibodies at 4 °C with shaking overnight. Following extensive washing with TBS-T, the membrane was incubated with secondary antibodies for 2 h. After washing with TBS-T, protein bands were visualized using the BS ECL Plus Kit (Biosesang, Seongnam, Republic of Korea) and photographed with the Chemidoc imaging system (Vilber Lourmat, France). Quantification of the detected bands was performed using ImageJ software (Version 1.54k, NIH, Bethesda, MD, USA), and the data were presented in graph form.

### 3.7. Primary Skin Irritation Test

A total of 33 healthy female volunteers aged between 21 and 54 years (mean age: 43.58 ± 9.29 years), with no history of irritant or allergic contact dermatitis, participated in this study. Squalene was used as the negative control. Voglibose was dissolved in squalene to prepare test solutions at concentrations of 50 and 100 μM. Primary skin irritation responses were evaluated based on the PCPC guidelines. The irritation response for squalene and voglibose in squalene was calculated using the formula provided below.

$$\mathrm{Response}=\frac{\sum \left(Grade\times No.of\;Responders\right)}{4(Maximum\;Grade)\times n(Total\;Subjects)}\times 100\times 1/2$$

This study was conducted in compliance with the ethical principles outlined in the Declaration of Helsinki and was approved by the Industrial Review Board (IRB) of Dermapro Inc. Written informed consent was obtained from all participants prior to the study (IRB number: 1-220777-A-N-01-DICN22080).

### 3.8. Statistical Analysis

The experimental results are presented as the mean ± standard deviation (SD) from three independent experiments. Statistical significance was determined using Student’s *t*-test. The significance levels are indicated as follows: #: *p* < 0.001 compared to the unstimulated control group. ***: *p* < 0.05, **: *p* < 0.01, and ***: *p* < 0.001 compared to the group treated with α-MSH alone.

## 4. Conclusions

This study explored the anti-melanogenic effects and molecular mechanisms of voglibose, highlighting its potential for repurposing as a therapeutic agent for hyperpigmentation disorders and the development of functional cosmetics. The results demonstrate that voglibose inhibited melanin synthesis and tyrosinase activity in a dose-dependent manner and reduced the expression of MITF, TRP-1, and TRP-2 proteins, effectively suppressing melanogenesis. Furthermore, voglibose modulated key signaling pathways, including PKA/CREB, MAPK, PI3K/AKT, and GSK3β/β-Catenin, and its topical safety was confirmed through human skin irritation tests. However, this study was conducted using the B16F10 melanoma cell model, and the effects of voglibose on primary human melanocytes, which provide a more physiologically relevant model for melanin biosynthesis, were not validated. Future studies are necessary to evaluate the efficacy of voglibose in primary human melanocytes and to confirm its long-term safety and effects on various skin types and with different environmental factors, such as UV exposure, through clinical trials. Additionally, further investigations should clarify the detailed molecular interactions observed in the signaling pathway analysis. Addressing these limitations will help expand the potential applications of voglibose and provide a stronger scientific foundation for its use in treating hyperpigmentation and developing functional cosmetics.

## Figures and Tables

**Table 1:** The results from the primary human skin irritation tests (*n* = 33).

No.	Sample	Respondents	20 min After Removal				24 h After Removal				Reaction Grade (R) *		
			+1	+2	+3	+4	+1	+2	+3	+4	24 h	48 h	Mean
1	Voglibose (50 μM)	0	-	-	-	-	-	-	-	-	0	0	0
2	Voglibose (100 μM)	0	-	-	-	-	-	-	-	-	0	0	0.0
3	Squalene	0	-	-	-	-	-	-	-	-	0	0	0

The reactions were assessed at 20 min and 24 h after the removal of the treatment by the investigator, according to the PCPC guidelines. * The range of irritation from “no to slight irritation”: 0.00 ≤ *R* < 0.87.

## Data Availability

All data generated or analyzed during this study are fully available within this published article.
